# Trends and Predictors of Venous Thromboembolism and Major Hemorrhagic Events in Hospitalized Leukemia Patients: A Cross-Sectional Analysis of the NIS (2016–2020)

**DOI:** 10.3390/clinpract15070117

**Published:** 2025-06-25

**Authors:** Daniel Antwi-Amoabeng, Bryce D. Beutler, Vijay Neelam, Mark Ulanja

**Affiliations:** 1 Department of Internal Medicine, Christus Ochsner St. Patrick Hospital, Lake Charles, LA 70602, USA; vijay.neelam@christushealth.org; 2 Department of Radiology, University of California, San Francisco, CA 94143, USA; bryce.beutler@ucsf.edu; 3 Taussig Cancer Center, Cleveland Clinic, Cleveland, OH 44195, USA; ulanjam@ccf.org

**Keywords:** venous thrombosis, major bleeding, leukemia, protein calories malnutrition, healthcare economics

## Abstract

Background/Objectives: Venous thromboembolism (VTE) and major hemorrhagic events are significant complications in hospitalized leukemia patients, but contemporary analyses of their epidemiology, predictors, and impact on clinical outcomes remain limited. Methods: We conducted a cross-sectional study using the National Inpatient Sample (NIS) database from 2016 to 2020. Hospitalized leukemia patients were identified using ICD-10 codes. Trends in the incidence of venous thromboembolism (VTE) and bleeding were assessed across the years, and multivariable logistic regression models were used to evaluate the predictors of VTE and bleeding. We assessed the influence thromboembolic and hemorrhagic complications on length of stay, cost, and mortality outcomes. Results: Among 430,780 leukemia hospitalizations, the overall incidence of VTE was 5.4% and remained stable throughout the study period (*p* = 0.09), while hemorrhagic events = 5.6%) showed a significant upward trend (*p* = 0.01). Cerebrovascular accidents, central venous catheter insertion, and protein calorie malnutrition (PCM) were significant predictors of both VTE and hemorrhage. PCM demonstrated a dose-dependent relationship with both complications. VTE was associated with a 33.5% increase in length of stay (LOS) and a 35% increase in cost of care (COC). Hemorrhage was associated with 23.2% increase in LOS and 32.6% increase in COC. Only hemorrhagic events were independently associated with increased mortality (adjusted OR 2.88, *p* < 0.001). Conclusions: The incidence of VTE in hospitalized leukemia patients has remained stable while hemorrhagic complications have increased significantly. Nutritional status represents a potentially modifiable risk factor for both VTE and bleeding complications. The competing risk between thrombosis and hemorrhage varies with age and nutritional status, suggesting the need for nuanced thromboprophylaxis strategies in this vulnerable population.

## 1. Introduction

Venous thromboembolism (VTE), which includes deep vein thrombosis (DVT) and pulmonary embolism (PE), is a major complication in hospitalized patients. Malignancy represents a major risk factor for VTE due to increased endothelial injury from inflammatory cytokines, excess secretion of procoagulant factors, and dysregulated activation of the coagulation cascade. Individuals with acute leukemia are at particularly high risk of VTE due to marked hyperviscosity and leukocytosis. Between 5 and 15% of patients with leukemia will develop VTE, most often within the first year of diagnosis [1,2]. There are additional VTE risk factors that are unique to the inpatient hospital setting, including reduced mobility, repeat venipuncture, and the placement of central venous catheters. The need for VTE prophylaxis therefore represents a priority for clinicians in the hospital environment.

Hemorrhagic complications represent an equally important concern in acute leukemia due to disease-related factors such as thrombocytopenia, platelet dysfunction, and coagulopathy. The risk for major bleeding is increased in the hospital setting due to medically necessary invasive procedures, chemotherapy-induced myelosuppression, and the use of anticoagulation treatment for VTE prophylaxis and management. The incidence of major hemorrhage among patients with acute leukemia ranges from 0.22 to 0.46 per 100 hospitalizations [3]. Major bleeding events range from gastrointestinal bleeding to life-threatening intracranial hemorrhage, significantly impacting patient outcomes and resource utilization.

The complex interplay between anticoagulation and hemorrhagic complications presents a clinical challenge in the treatment of patients with acute leukemia. Clinicians must carefully balance thromboprophylaxis and bleeding risk to prevent avoidable complications and optimize patient outcomes. Although VTE prevention and treatment strategies have evolved, the contemporary evaluation of VTE incidence, trends, and risk factors in hospitalized leukemia patients and the accompanying hemorrhagic complications remains limited. In this cross-sectional study, we use the National Inpatient Sample (NIS) database (2016–2020) to provide an updated analysis of VTE and bleeding event epidemiology and trends in hospitalized leukemia patients. The study further assesses the association between thromboembolic and hemorrhagic complications, with the length of stay, the cost of care, and inpatient mortality as secondary outcomes. The study examines trends and risk factors to inform clinical practice and guide thromboprophylaxis strategies in this vulnerable population.

## 2. Materials and Methods

### 2.1. Study Population and Data Elements

We extracted data on discharge encounters from the NIS from 2016 through 2020. We used the appropriate International Classification of Diseases, Tenth Revision, Clinical Modification (ICD-10-CM) codes for diagnoses and applied the International Classification of Diseases, Tenth Revision, Procedure Coding System (ICD-10-PCS) codes for inpatient procedures to create the cohort of interest for this study [4]. The ICD-10-CM codes and ICD-10-PCS codes used for this study have been described previously [5]. Hospitalized patients with leukemia were identified using ICD-10-CM codes for acute lymphoblastic leukemia (C91.0x), acute myeloid leukemia (C92.0x), and other specified leukemias (e.g., C93.0x, C94.0x, C95.0x). Venous thromboembolism (VTE) was defined using codes for deep vein thrombosis (I82.0xx) and pulmonary embolism (I26.0xx). Major hemorrhagic events included gastrointestinal bleeding (K92.2, K25.0, K25.4, etc.), intracranial hemorrhage (I61.0x, I62.0x), and pulmonary hemorrhage (R04.2). Central venous catheter insertion was identified using ICD-10-PCS codes such as 05HN33Z and related procedural codes. We defined major hemorrhagic events as a composite of intracranial hemorrhage, pulmonary hemorrhage, and gastrointestinal bleeding. The NIS is the largest publicly available all-payer inpatient care survey database in the United States. It was developed for the Healthcare Cost and Utilization Project (HCUP); it is sponsored by the Agency for Healthcare Research and Quality (AHRQ) and systematically samples approximately 20% of all hospitalizations in the United States, covering over 97% of the nation [4].

### 2.2. Statistical Analyses

Continuous data were summarized as median values with the interquartile range or mean values with standard deviation, where applicable, and categorical variables were derived as counts expressed as a percentage of the study sample. We compared the distribution of baseline characteristics and outcomes between patients who had VTE and bleeding and those who did not, using the Chi-squared test for categorical variables and the Wilcoxon Rank Sum test to obtain equality of the means for continuous variables. We used logistic regression to model trends in VTE and bleeding across years (as a continuous variable) and assessed trend significance using the Jonckheere–Terpstra test for trends. In post hoc analyses, we then used marginal effects to generate predicted probabilities of VTE and bleeding by age across defined classes of malnutrition. We then used multinomial logistic regression to assess the competing risk of VTE and hemorrhage for each malnutrition class. To assess the predictors of VTE and hemorrhagic complications, we performed univariate logistic regression analysis, using patient- and hospital-level characteristics, and included covariates with statistical significance and/or those deemed to have clinical significance in a base multivariable model of the odds of the outcomes. We selected the most parsimonious model using manual stepwise elimination of covariates and ensured model fitness with appropriate post-estimation tests. We applied a similar approach to assess the predictors of length of stay, cost of care, and all-cause inpatient mortality. Encounters with patients of an age less than 18 years old and observations with missing data for age, sex, and race were excluded from the analyses. To account for the complex nature of the NIS survey database, appropriate survey weights were applied where appropriate as recommended by the HCUP. All analyses were performed at a two-tailed 5% level of significance using Stata version 18.0 (Stata Corporation, College Station, TX, USA).

## 3. Results

### 3.1. Baseline Characteristics and Primary Outcomes

There were 86,202 unweighted discharge encounters involving leukemia, corresponding to a nationally representative weighted estimate of 430,780 hospitalizations after the application of survey weights. Table 1 summarizes the baseline characteristics of subjects who experienced venous thromboembolism (VTE) and those who experienced hemorrhagic events compared to those who did not. There were 23,205 encounters involving VTE in the study period, representing 5.4% of the survey-weighted sample. There were no differences in the distribution of VTE among males and females (*p* = 0.81). The median age of encounters with VTE was slightly lower than that of those without VTE (60 vs. 61 years, *p* < 0.001); however, VTE events were more prevalent among patients aged 60–69 and ≥80 years, suggesting that there was a non-linear association between age and VTE risk. There were no differences in income distribution (*p* = 0.31) and insurance status differences were minimal. Analysis of hospital-level characteristics showed that significantly more VTE occurred in large-sized hospitals (72.9% vs. 69.1%, *p* < 0.001) and that it was more common in large-size hospitals in urban teaching hospitals (92.5% vs. 88.3%, *p* < 0.001). Comorbid conditions that showed significant associations with VTE occurrence included atrial fibrillation (6.9% vs. 5.9%), CVA (2.6% vs. 1%), heart failure (14.4% vs. 13.1%), and obstructive sleep apnea (7.5% vs. 6.5%). Protein calorie malnutrition (PCM) and obesity showed a dose-dependent association, where there were significantly more VTE events in the more severe disease state.

There were 24,300 encounters with discharge codes for major hemorrhagic events, representing 5.6% of the survey-weighted sample. There were no significant differences in the distribution of sex between those who had bleeding and those who did not (*p* = 0.39) (Table 1). There were significantly more bleeding episodes in the older age groups (*p* < 0.001), measured from 60 years at 10-year increments. Subjects who experienced bleeding were significantly older than those who did not: median age, years (IQR) 60 (43–71) vs. 65 (52–74), *p* < 0.001. Income level did not affect the distribution of bleeding episodes. However, significantly more patients had bleeding events among Medicare patients (50.9% vs. 41.9%, *p* < 0.001) than other types. Hospital-level characteristics did not influence the incidence of bleeding, i.e., *p* > 0.05 for bed size, location, and hospital teaching status. Among comorbid conditions, atrial fibrillation (7.3% vs. 5.9%), COPD (15.4% vs. 14.1%), CVA (3.5% vs. 0.9%), heart failure (16.9% vs. 13%), hypertension (25.3% vs. 21.3%), and renal disease (13.7% vs. 11.7%), any degree of protein calorie malnutrition was associated with a higher incidence of bleeding, *p* < 0.05. The relationship between weight and bleeding is less linear compared to that seen for VTE.

We identified and analyzed several acute inpatient clinical events and procedures that potentially influence the occurrence of VTEs or hemorrhagic complications and summarized their distribution in Table 2. Patients who developed VTE showed significantly higher rates of central venous catheter (CVC) insertion (33.4% vs. 18.1%, *p* < 0.001) and CVC infection (5.3% vs. 2.5%, *p* < 0.001). While CVC insertion was associated with significantly higher rates of major hemorrhage (27.3% vs. 18.5%, *p* < 0.001), CVC infection rates did not differ by hemorrhage status. Predictably, thrombocytopenia was more common in those who had major bleeding episodes (31.8% vs. 22.9%, *p* < 0.001). Acute kidney injury, acute liver injury, invasive mechanical ventilation, sepsis, and septic shock were significantly associated with higher VTE and bleeding rates (Table 2). Receiving chemotherapy or immunotherapy was associated with significantly lower rates of VTE and hemorrhage.

### 3.2. Acute Inpatient Events and Secondary Outcomes

The event rates for the secondary outcomes and acute inpatient events of importance to this study are summarized in Table 2. Patients who developed VTE had significantly longer hospital stays, with a median of 11.5 days (interquartile rage: 5–27 days) compared to 5 days (IQR: 3–13 days) for those without VTE (*p* < 0.001). Similarly, patients who had hemorrhagic complications had extended hospital stays, with a median of 10 days (IQR: 4–26 days) versus 5 days (IQR: 3–13 days) for those without bleeding events (*p* < 0.001). The cost of care rounded to the nearest US dollar was significantly higher among those with VTE or bleeding complications. Those who had VTE were charged approximately 2.2 times more compared to those without VTE, USD 46,872 (IQR: USD 16,473–USD 125,724), *p* < 0.001. Subjects with hemorrhagic complication were billed twice as much as those without major bleeding episodes USD 43,663 (IQR: USD 15,287–USD 126,279), *p* < 0.001. The cost of care represents the total charge for the care received in the hospital and not necessarily how much the patient paid for that care. It was adjusted for variations across hospitals and clinical conditions using conversion factors provided by the HCUP. The all-cause inpatient mortality rate was higher in those with thromboembolic and bleeding complications. The overall mortality rate for encounters with acute leukemia was 7.1%. From Table 2, the excess mortality with VTE was calculated as 3.3% and the proportion of total mortality attributable to VTE was 2.5%. Similarly, for those with hemorrhagic complications, the excess mortality with bleeding was 17.9%, with 14.1% of the total mortality attributable to bleeding complications.

### 3.3. Trends in VTE and Hemorrhagic Complications

**Figure 1:**
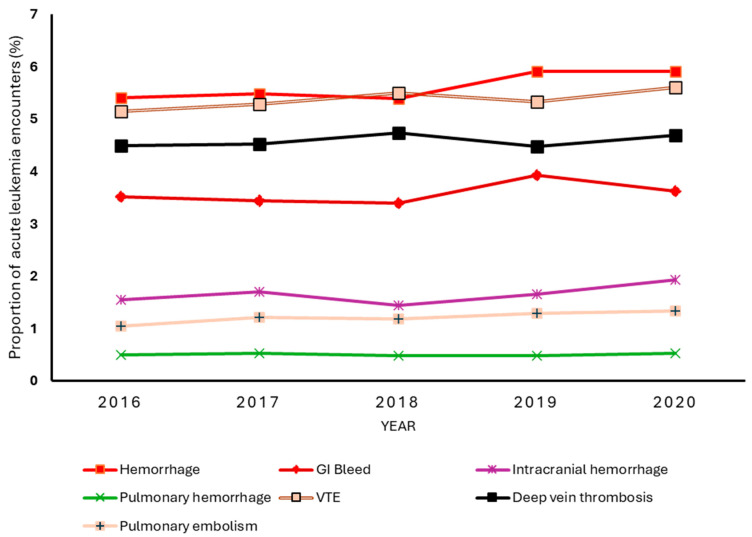
Temporal trends in venous thromboembolism (VTE) and hemorrhagic complications among hospitalized acute leukemia patients, 2016–2020. VTE is composite of deep vein thrombosis and/or pulmonary embolism. Bleeding comprises gastrointestinal (GI) bleeding, intracranial hemorrhage, and pulmonary hemorrhage.

Figure 1 shows the year-to-year rate of VTE and bleeding events among leukemia patients in the study sample. The overall incidence of VTE was 5.4%, with the small year-to-year fluctuations ranging from 5.2% in 2016 to 5.6% in 2020. However, no statistically significant monotonic trend was observed over the study period, as assessed by the Jonckheere–Terpstra test (*p* = 0.09). Deep vein thrombosis occurred more frequently than pulmonary embolism. The incidence of bleeding was 5.6%. Overall, bleeding events showed a slight upward trend, particularly from 2018 to 2019. The test for trends showed an increasing linear trend in hemorrhagic complications from 2016 to 2020, *p* = 0.01. Gastrointestinal bleeding was the most common hemorrhagic complication, with an overall rate of 3.7%, which remained stable despite the slight uptick in rates from 2018 to 2019. Interestingly, despite being a less common bleeding complication with an overall incidence of 1.7%, intracranial hemorrhage showed a significant upward linear trend over time, *p* = 0.01. Thus, the increase in overall bleeding rates from 5.4% to 5.9% was primary driven by increases in intracranial hemorrhage. Pulmonary hemorrhage remained the least common event, with an insignificant linear trend.

### 3.4. Predictors of VTE and Hemorrhagic Complications

**Figure 2:**
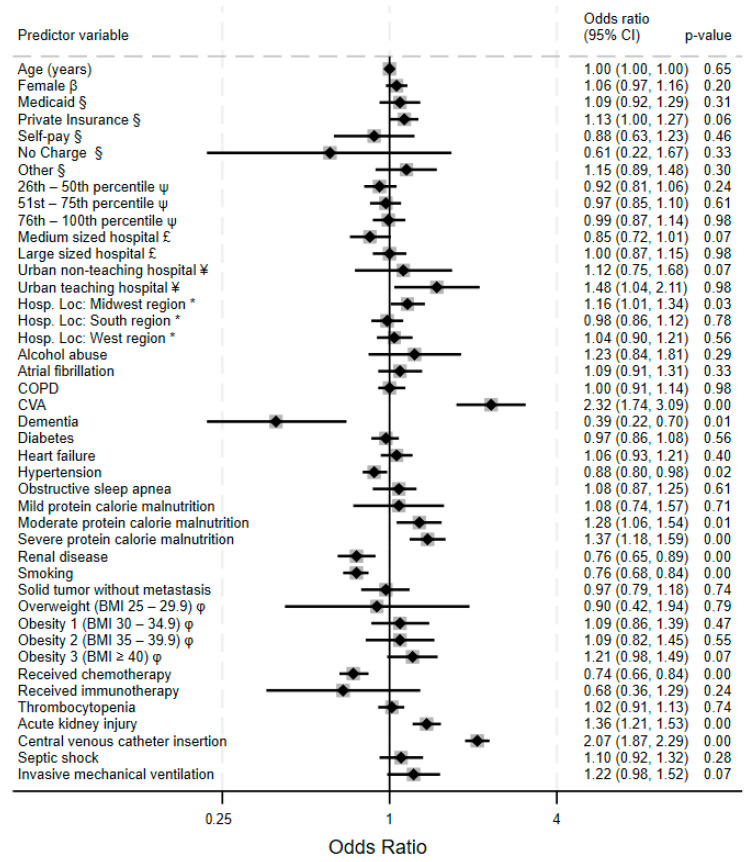
Forest plot showing adjusted odds ratio (95% confidence interval) for venous thromboembolism for various predictor variables in multivariable logistic regression model. Key: β = compared to males; § = compared to Medicare; ψ = compared to subjects with zip codes in the 0–25th percentile of US household income; £ = compared to small-bed-size hospital; ¥ = compared to rural community hospitals; * = compared to northeastern region of the United States; φ = compared to normal BMI 18.5–24.5. Legend: COPD: chronic obstructive pulmonary disease apnea; CVA = cerebrovascular accident; BMI = body mass index.

Figure 2 displays the result of a multivariate analysis of VTE predictors in acute leukemia patients. Cerebrovascular accident had the highest odds of VTE (adjusted odds ratio, OR 2.32, 95% confidence interval (CI) 1.74–3.09, *p* < 0.001), followed by central venous catheter insertion (OR 2.07, 95% CI 1.87–2.29, *p* < 0.001), likely reflecting a shared pathophysiological mechanism of vascular injury and hypercoagulability. Protein calorie malnutrition shows a stepwise gradient of increasing risk, with the odds ratios escalating from mild (OR 1.08, non-significant) to moderate (OR 1.28, 95% CI 1.06–1.54, *p* = 0.01) to severe protein calorie malnutrition (OR 1.37, 95% CI 1.18–1.59, *p* < 0.001), suggesting the existence of a linear relationship between malnutrition and thromboembolic tendencies. Odds for VTE were higher at urban teaching hospitals.

**Figure 3:**
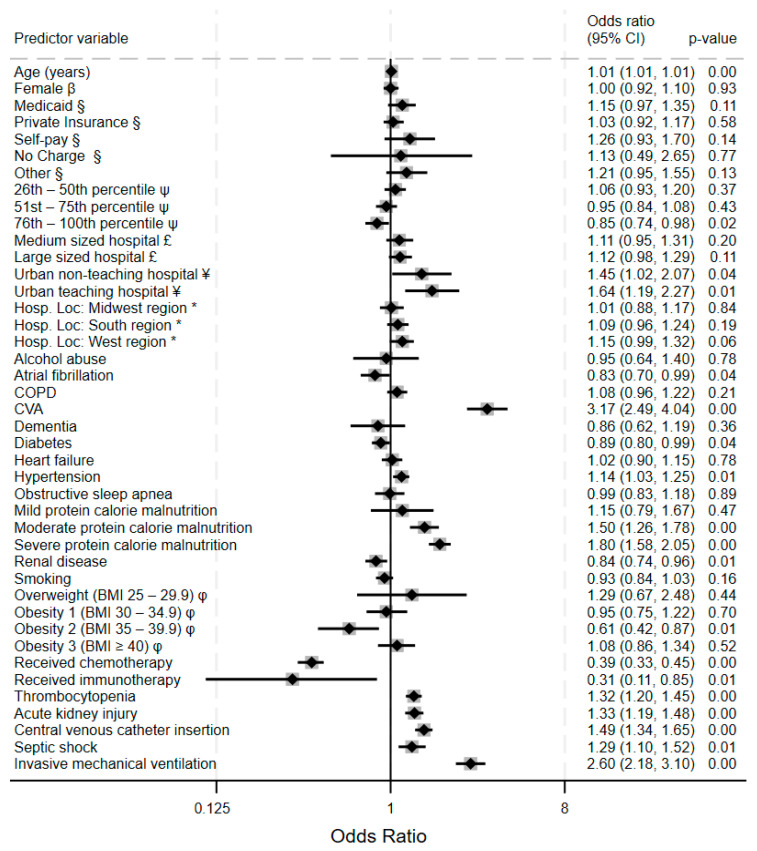
Forest plot showing adjusted odds ratio (95% confidence interval) for hemorrhagic events for various predictor variables in the multivariable logistic regression model. Key: β = compared to males; § = compared to Medicare; ψ = compared to subjects with zip codes in the 0–25th percentile of US household income; £ = compared to small-bed-size hospital; ¥ = compared to rural community hospitals; * = compared to northeastern region of the United States; φ = compared to normal BMI 18.5–24.5. Legend: COPD: chronic obstructive pulmonary disease apnea; CVA = cerebrovascular accident; BMI = body mass index.

Analysis of the risk factors for hemorrhagic events among acute leukemia patients reveals several significant predictors. Figure 3 is a forest plot showing that the relative strengths of the cerebrovascular accident demonstrates the strongest association with major bleeding (OR) 3.17, 95% CI 2.49–4.04, *p* < 0.001), followed by invasive mechanical ventilation (OR 2.60, 95% CI 2.18–3.10, *p* < 0.001). Central venous catheter insertion is associated with significantly higher odds of bleeding (OR 1.49, 95% CI 1.34–1.65, *p* < 0.001). Protein calorie malnutrition (PCM) exhibits a striking dose–response relationship with bleeding risk, where severe PCM (OR 1.80, 95% CI 1.58–2.05) and moderate PCM (OR 1.50, 95% CI 1.26–1.78) have substantially increased bleeding risk while mild PCM shows no significant association. On the other hand, excess calories expressed as obesity classes by BMI show a non-linear, complex relationship between body mass and major bleeding events in leukemia patients: class 2 obesity shows a protective effect (OR 0.61, 95% CI 0.42–0.87, *p* = 0.01), while the other obesity BMI categories lack significant associations. Urban teaching hospitals demonstrate significantly higher hemorrhage rates (OR 1.64, 95% CI 1.19–2.27, *p* = 0.01) compared to non-teaching facilities, possibly reflecting more case complexity.

### 3.5. The Influence of Malnutrition on VTE and Hemorrhagic Risks

**Figure 4:**
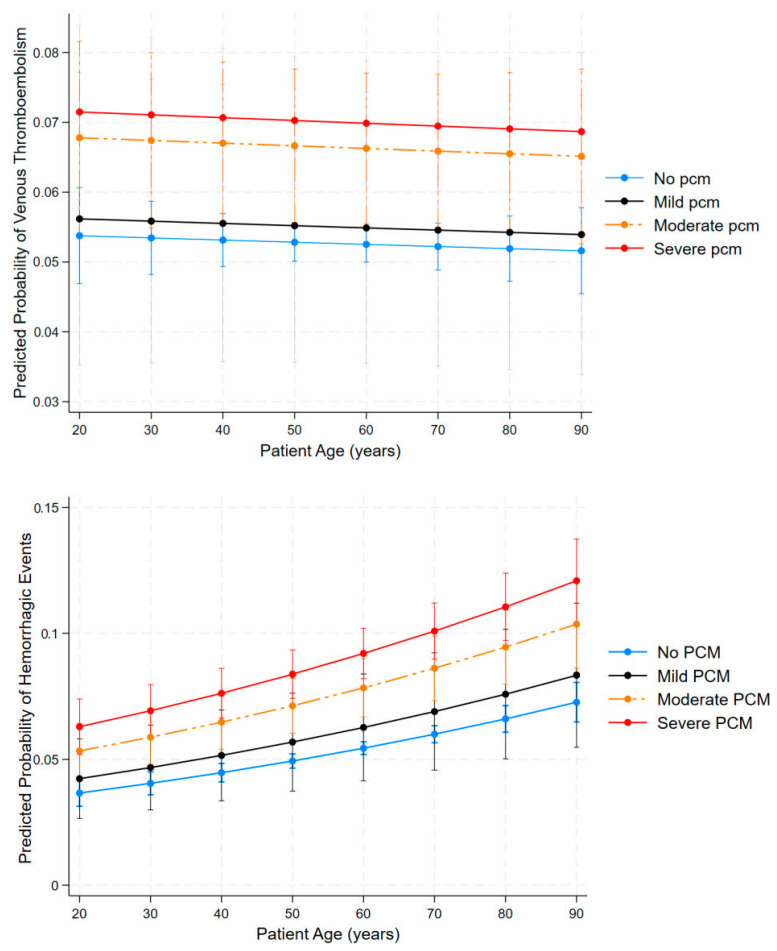
Predicted probabilities of venous thromboembolism (**top panel**) and hemorrhagic events (**bottom panel**) by age and protein calorie malnutrition (PCM) status in hospitalized acute leukemia patients. Predictions are derived from multivariable logistic regression model adjusted for the covariates. Error bars represent 95% confidence interval.

To further explore the complex relationship between age, malnutrition status, and the primary outcomes, we conducted post hoc margins analysis to estimate predicted probabilities across patient subgroups while holding other covariates at their mean values. Figure 4 shows the margins plot of the relationship. The top panel shows the predicted probability of VTE by age across different protein calorie malnutrition (PCM) categories. VTE probability modestly decreases with advancing age across all malnutrition status categories, in contrast to findings in the general population, where thrombotic risk increases with age. The nutritional gradient remains consistent throughout the age spectrum, with severe PCM maintaining approximately 40% higher VTE risk (~7%) compared to well-nourished patients (~5%). Moderate PCM demonstrates an intermediate risk profile, supporting the dose-dependent relationship between malnutrition and thromboembolic tendencies.

The bottom panel demonstrates the predicted probability of hemorrhagic events by age for all PCM classifications. In contrast to VTE, bleeding risk increases substantially with age across all PCM categories. Increasing age consistently elevates bleeding risk across all PCM categories from age 20 to 90, and worsening malnutrition progressively increases risk, with severe PCM cases showing a substantially higher probability of hemorrhage than those without PCM across all age groups. Elderly subjects (≥ 80 years) with severe PCM face a hemorrhagic risk of more than 12%, which is nearly threefold higher compared to young, well-nourished patients (~4%). This gradient remains consistent across the age spectrum, with malnutrition severity consistently stratifying risk. Taken together, these findings suggest there is a complex interaction between age and nutritional status, shaping thrombohemorrhagic risk. VTE risk appears to disproportionately affect younger, malnourished patients, while hemorrhagic risk escalates with age and PCM severity.

### 3.6. Influence of Malnutrition on the Risk for VTE and/or Major Bleeding

We employed multinomial logistic regression methods to address the competing risk of VTE and hemorrhage in hospitalized acute leukemia patients, with a focus on the role of malnutrition. Table 3 shows the nuanced relationship between malnutrition severity and thrombohemorrhagic complications in our patient group. Both VTE and hemorrhagic risks progressively increase with worsening malnutrition status and the association is more robust in severe disease as evidenced by the trend from non-significant relationships in mild PCM to very strong statistical significance in severe PCM. Hemorrhagic events have a stronger association with malnutrition than thromboembolic events, with severe PCM having an increased risk of hemorrhage only by 78% (RRR 1.73, 95% CI 1.50–1.99, *p* < 0.001) compared to a 28% increase in VTE-only risk (RRR 1.28, 95% CI 1.08–1.51, *p* < 0.001). There is a substantial increase in risk for both complications simultaneously among subjects with more serious malnutrition status: severe PCM nearly triples the risk (RRR 2.88) while moderate PCM doubles the risk (RRR 2.00). There appears to be a threshold effect of malnutrition as mild PCM shows no statistically significant associations with any outcome category. Thus, the deleterious effect of malnutrition is only apparent in moderate-to-severe stages of malnutrition.

### 3.7. Predictors of Length of Stay and Cost of Care

**Figure 5:**
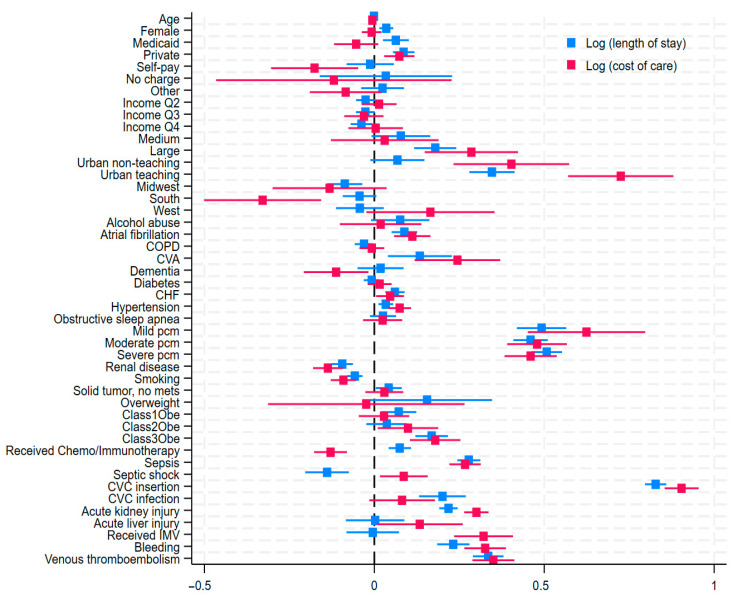
Predictors of hospital length of stay and cost of care in acute leukemia in multivariable linear regression analysis. The blue squares represent coefficients for log-transformed length of stay; red squares represent coefficients for log-transformed cost. Horizontal lines indicate 95% confidence intervals. Values greater 0 indicated increased length of stay or cost; values less than 0 indicate decreased length of stay or cost. Multiplying the coefficient by 100 provides an approximate percentage change in the value of interest. Base comparators are previously described in Figure 2. IMV = invasive mechanical ventilation. CVC = central venous catheter. CHF = congestive heart failure.

Both thromboembolic and hemorrhagic events were associated with increased length of stay (LOS) and cost of care (COC). In both instances, subjects had at least twice as long median hospital stays, and they were billed at least twice as much compared to those without thrombohemorrhagic complications. Figure 5 summarizes the influence of covariables on LOS and COC. In multivariable linear regression analysis, VTE was associated with a 33.5% increased LOS and a 35% increased COC (coefficient, 0.33, 95% CI: 0.29–0.38) and (Coeff, 0.35, 95% CI: 0.29–0.41, *p* < 0.001), respectively. Bleeding was associated with a 23.2% increase in LOS (Coeff, 0.23, 95% CI: 0.18–0.28, *p* < 0.001) and a 32.6% increase in COC (Coeff, 0.32, 95% CI: 0.26–0.39, *p* < 0.001). The insertion of central venous catheters has the highest impact on both LOS and COC, with more than a 0.8-log-unit increase in both parameters, attaining values of Coeff 0.91 (0.86–0.96, *p* < 0.001) and Coeff 0.84 (0.81–0.87, *p* < 0.001), respectively. This intervention was also associated with increased odds of VTE and hemorrhage. Acute kidney injury, which was also associated with increased odds of VTE, and hemorrhage show significant associations with increased LOS and COC.

### 3.8. Predictors of Inpatient Mortality

**Figure 6:**
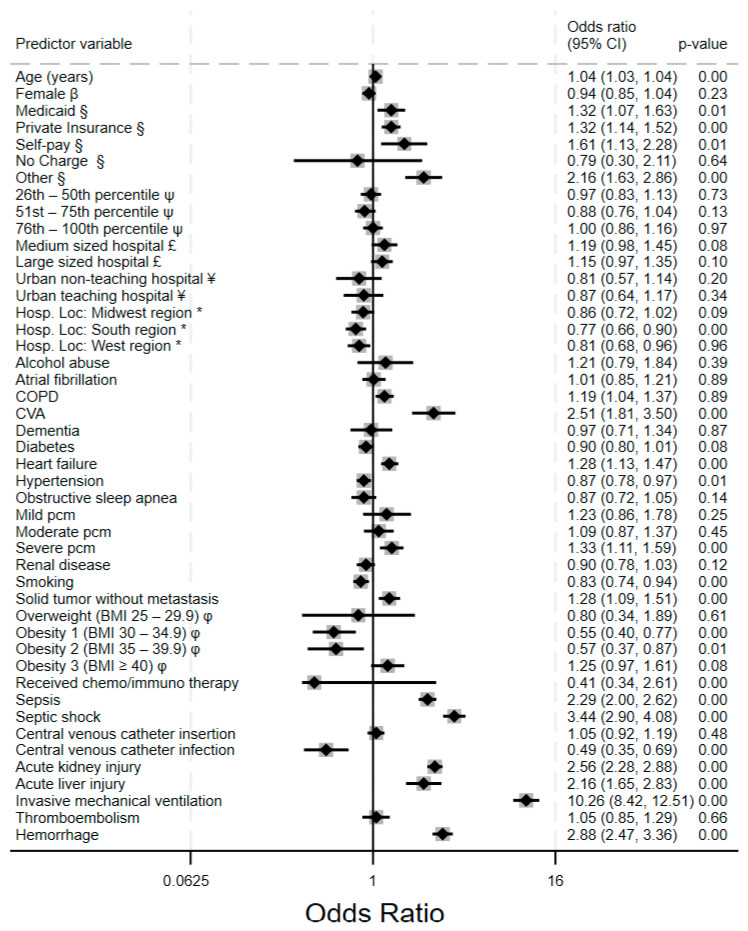
Forest plot of adjusted odds ratios for inpatient mortality predictors among hospitalized acute leukemia patients. All estimates adjusted for demographics, hospital characteristics, comorbidities, and clinical complication. Key: β = compared to males; § = compared to Medicare; ψ = compared to subjects with zip codes in the 0–25th percentile of US household income; £ = compared to small-bed-size hospital; ¥ = compared to rural community hospitals; * = compared to northeastern region of the United States; φ = compared to normal BMI 18.5–24.5. Legend: COPD: chronic obstructive pulmonary disease apnea; CVA = cerebrovascular accident; BMI = body mass index; PCM = protein calorie malnutrition.

Invasive mechanical ventilation was associated with the highest odds of inpatient mortality (aOR 10.26, 95% CI: 8.42–12.51), demonstrating a strong association with increased mortality, though this does not imply a direct tenfold increase in absolute risk. The adjusted odds of VTE and hemorrhage were 1.05 (a modest increase) and 2.88, respectively. However, the aOR for VTE did not reach statistical significance (*p* = 0.66). In Figure 6, other significant risk factors of mortality include sepsis and septic shock (2.29, 2.00–2.26 and 3.44, 2.90–4.08, respectively), acute kidney injury (2.58, 2.28–2.88), and acute liver injury (2.16, 1.65–2.83), as well as cerebrovascular accidents (2.51, 1.81–3.50). Patients with severe protein calorie malnutrition have significantly higher odds of mortality (1.11, 1.33–1.59). Patients who are healthy enough to receive chemo-/immune therapy had significantly lower odds of death (aOR 0.41, 0.34–2.61); however, this association did not reach statistical significance. Obesity appears to have a protective effect, as those with class 1 and class 2 obesity had significantly lower adjusted odds of death compared to those without obesity (aOR 0.55 and 0.57, respectively).

## 4. Discussion

Individuals with acute leukemia face a high risk of both VTE and hemorrhagic complications. These risks are heightened in the inpatient setting secondary due to decreased mobility, the administration of induction chemotherapy, and medically necessary invasive procedures. The existing prophylaxis strategies have been moderately effective in preventing VTE among hospitalized patients. However, little progress has been made in VTE prevention over the past decades. In an analysis of California Cancer Registry data collected between 1993 and 1999, the two-year cumulative incidence of VTE among patients with acute leukemia was 5.2% [6]. A subsequent study of NIS data collected between 2011 and 2015 showed little improvement; the VTE rate among patients hospitalized with acute leukemia ranged between 6.0 and 6.6% [7]. More recent meta-analyses and systematic reviews described similar findings [1]. In our analysis, the overall incidence of VTE ranged from 5.2% to 5.6%, with no significant change over the course of the five-year study period.

There have been remarkable advancements in hematology and oncology over the past thirty years. Imatinib was developed for the treatment of chronic myeloid leukemia in the late 1990s [8]. The first immune-checkpoint inhibitors were introduced just over a decade later in 2011 [9]. Chimeric antigen receptor T cell (CAR-T) therapy was recently approved by the Food and Drug Administration and is now widely used at community and academic centers across the country [10]. However, during this period of unprecedented medical progress and biomedical innovation, the incidence of VTE failed to improve. There is a strong association between VTE and cost of care, length of stay, and mortality [11]. The effect of VTE on mortality is particularly pronounced among patients with an underlying malignancy [12]. The development of new strategies to reduce the rate of VTE therefore has the potential to make a meaningful impact on clinical outcomes.

The risk of VTE and hemorrhage exists in a state of dynamic equilibrium; aggressive pharmacologic VTE prophylaxis is inextricably linked with an increased risk of bleeding. In our analysis, there was a small but significant upward trend in bleeding events over the course of the study period, with an overall increase in bleeding rates from 5.4% in 2016 to 5.9% in 2020. The increase in intracranial hemorrhages we observed over time may reflect a combination of factors: an aging leukemia inpatient population with a higher comorbidity burden; increased use of anticoagulants known to elevate ICH risk; and more frequent application of neuroimaging, leading to enhanced case detection. These findings echo those of Lioutas et al., who observed stable age-adjusted ICH incidence from 1948 to 2016, with a notable increase among adults aged ≥ 75 years coinciding with a threefold rise in anticoagulant use—suggesting a temporal, though not necessarily causal, relationship [13]. The approach to reducing VTE rates should therefore emphasize medical optimization and the mitigation of modifiable risk factors rather than the increased use of anticoagulants. In our analysis, there was a stepwise gradient of increasing risk associated with PCM and both VTE and hemorrhage. Rates of VTE in patients with mild, moderate, and severe PCM were 1.1%, 5.2%, and 9.6%, respectively. Rates of hemorrhage in patients with mild, moderate, and severe PCM were 1.3%, 6.1%, and 11.5%, respectively. PCM therefore appears to represent a promising target for intervention, potentially reducing both VTE and bleeding complications among hospitalized patients. Severe protein calorie malnutrition contributes to both thrombotic and bleeding complications through a combination of endothelial dysfunction, systemic inflammation, and impaired synthesis of clotting factors. This results in a paradoxical state of increased clotting and bleeding susceptibility in hospitalized patients. The routine assessment of serum prealbumin on admission could be useful for identifying patients at risk of PCM [14]. Revising coding and billing for PCM may improve reimbursement and incentivize aggressive interventions in the inpatient setting [15]. In addition, the measures of integrating registered dietitian nutritionists into the medical team and implementing order-writing privileges have been shown to improve patient nutrition status [16]. Nutritional supplementation and serum prealbumin and albumin monitoring could be performed at low cost and significantly improve outcomes.

Other factors that influenced both VTE and bleeding risk to varying degrees included cerebrovascular accident, central venous catheter insertion, and invasive mechanical ventilation. A tool incorporating these factors to provide a semiquantitative analysis of relative risk would be useful to guide clinical decision-making. Although several algorithms have been developed to assess the risk of bleeding among patients on anticoagulation, including the VTE-BLEED score, there are no comprehensive risk stratification constructs that integrate multiple competing risk factors for both VTE and hemorrhage. The results of this analysis, among others, provide important foundational data that could be used to improve patient management.

The small but significant increase in hemorrhage in the setting of stable VTE rates suggests that routine inpatient VTE prophylaxis protocols should be revisited. However, due to the absence of inpatient medication data in the NIS, we were unable to evaluate the influence of anticoagulant use or changes in anticoagulation practices on bleeding trends over time. It has been definitively established that low-molecular-weight heparin (LMWH) is more effective and associated with decreased risk of bleeding complications relative to unfractionated heparin, and most hospital systems and healthcare networks now use LMWH as the standard anticoagulant agent in the inpatient setting [17,18,19]. However, emerging evidence indicates that direct oral anticoagulants (DOACs) may represent a suitable alternative to LMWH. In a 2014 meta-analysis by Castellucci et al., the authors reported that DOACs were associated with a lower bleeding risk as compared to LMWH among patients undergoing therapeutic anticoagulation for VTE [20]. A subsequent analysis by Kapoor et al. showed that DOACs may also be appropriate for VTE prophylaxis. The Kapoor group assessed the relative risk of VTE versus hemorrhage among patients requiring VTE prophylaxis following orthopedic surgery and found that DOACs had a significantly more favorable VTE-to-hemorrhage profile relative to LMWH [21]. In addition to a lower risk of bleeding, DOACs have been shown to be more efficacious and cost-effective than LMWH for the treatment of cancer-associated thrombosis [22,23]. Moreover, patients strongly prefer and demonstrate better adherence to DOACs as compared to LMWH [24]. Although some data suggest that DOACs may be associated with a slightly increased risk of non-major bleeding relative to LMWH [25], the preponderance of the evidence indicates that DOACs offer greater effectiveness than LMWH with a similar or lower risk of hemorrhage [26]. Future large-scale randomized-controlled trials comparing DOACs and LMWH would be of value to clarify the most appropriate inpatient pharmacologic VTE prophylaxis strategy. Novel factor XI(a) inhibitors also show a great deal of promise for VTE prophylaxis and warrant further investigation [27].

There are several limitations to this study, inherent to its design. The study was retrospective and therefore causation could not be assessed. In addition, the NIS database does not include information pertaining to VTE prophylaxis; it cannot be determined if all hospitals included in the dataset used routine VTE prophylactic strategies. The relationship between obesity and bleeding outcomes in our sample was somewhat paradoxical, with class 2 obesity demonstrating a significant protective effect. Previous data show that obesity increases bleeding risk among patients on VTE prophylaxis and it is conceivable that the protective effect observed in our analysis represented statistical noise [28]. Nevertheless, despite these limitations, the findings presented herein provide important information related to VTE and bleeding risk among patients hospitalized with leukemia. Future large-scale studies are warranted to develop risk stratification tools and determine optimal VTE prophylaxis strategies.

## 5. Conclusions

Our analysis reveals the complex interplay between thrombotic and hemorrhagic risks in hospitalized leukemia patients. While VTE rates remained stable from 2016 to 2020, hemorrhagic events increased significantly, especially intracranial hemorrhage. This study identifies protein calorie malnutrition as a critical modifiable risk factor with a dose-dependent association with both VTE and bleeding. We also identified a critical age-dependent shift in the thrombosis–hemorrhage balance: younger patients with malnutrition face higher VTE risks, while elderly malnourished patients demonstrate elevated hemorrhagic risk, suggesting the need for age-stratified thromboprophylaxis approaches. The findings from our study have immediate clinical implications, and we suggest that nutritional optimization may represent a cost-effective intervention to simultaneously reduce both VTE and bleeding complications in this vulnerable population. Further research focused on developing risk assessment tools should incorporate the competing thrombotic and hemorrhagic risks identified in this study, in particular accounting for the role of nutrition and age.

## Figures and Tables

**Table 1:** Baseline characteristics of hospitalized acute leukemia encounters stratified by venous thromboembolism (VTE) and hemorrhagic outcome status. Values are presented as absolute counts with corresponding percentages in parentheses, representing proportion of patients within each category who experienced VTE or hemorrhagic events.

Variable	VTE Absent 407,575 (94.6%)	VTE Present 23,205 (5.4%)	*p*-Value	Hemorrhage Absent 406,710 (94.4%)	Hemorrhage Present 24,300 (5.6%)	*p*-Value
**Age Categories (years)**			<0.001			<0.001
18–39	85,560 (21.0)	4755 (20.5)		87,025 (21.4)	3290 (13.5)	
40–49	43,425 (10.7)	2705 (11.7)		43,965 (10.8)	2165 (8.9)	
50–59	66,095 (16.2)	3990 (17.2)		66,505 (16.4)	3580 (14.7)	
60–69	93,805 (23.0)	6210 (26.8)		93,800 (23.1)	6215 (25.6)	
70–79	80,925 (18.9)	4135 (17.8)		79,065 (19.4)	5995 (24.7)	
≥80	37,965 (9.3)	1415 (17.8)		36,325 (8.9)	3055 (12.6)	
**Sex**			0.81			0.39
Male	226,115 (55.5)	12,830 (55.3)		225,315 (55.4)	13,630 (56.1)	
Females	181,460 (44.5)	10,375 (44.7)		181,170 (44.6)	10,665 (43.9)	
**Insurance Type**			<0.001			<0.001
Medicare	173.385 (42.6)	9180 (39.6)		170,205 (41.9)	12,360 (50.9)	
Medicaid	62,605 (15.4)	3585 (15.5)		63,090 (15.5)	3100 (12.8)	
Private insurance	146,340 (35.9)	9070 (39.1)		148,020 (36.4)	7390 (30.5)	
Self-pay	10,575 (2.6)	540 (2.3)		10,530 (2.6)	585 (2.4)	
No charge	1395 (0.3)	65 (0.3)		1400 (0.3)	60 (0.2)	
Other	12,985 (3.2)	745 (3.2)		12,955 (3.2)	775 (3.2)	
**Household Income**			0.31			0.13
0–25th percentile	96,090 (23.9)	5210 (22.8)		95,330 (23.8)	5970 (25.0)	
26th–50th percentile	99,670 (24.9)	5875 (25.7)		99,455 (24.9)	6090 (25.5)	
51st–75th percentile	104,105 (25.9)	6025 (26.3)		104,125 (26.0)	6005 (25.1)	
76th–100th percentile	101,245 (25.2)	5765 (25.2)		101,170 (25.3)	5840 (24.4)	
**Hospital Bed Size**			<0.001			0.94
Small	47,730 (11.7)	2595 (11.2)		47,510 (11.7)	2815 (11.6)	
Medium	788,220 (19.2)	3685 (15.9)		77,325 (19)	4580 (18.9)	
Large	281,850 (69.1)	16,930 (72.9)		281,875 (69.3)	16,905 (69.6)	
**Hospital Location**			<0.001			0.05
Rural	10,655 (26.1)	350 (1.5)		10,520 (25.9)	485 (2.0)	
Urban non-teaching	37,235 (9.1)	1390 (6.0)		36,445 (9.0)	2180 (9.0)	
Urban teaching	359,910 (88.3)	21,470 (92.5)		359,745 (8.8)	21,635 (8.9)	
**Hospital Region**			0.18			0.43
Northeast	79,385 (19.5)	4655 (20.1)		79,120 (19.5)	4920 (20.3)	
Midwest region	88,210 (21.6)	5175 (22.3)		88,250 (21.7)	5135 (21.1)	
South region	152,220 (37.3)	8210 (35.4)		151,285 (37.2)	9145 (37.6)	
West region	87,985 (21.6)	5170 (22.3)		88,055 (21.7)	5100 (21.0)	
**Comorbid Conditions**						
Alcohol abuse	2170 (1.2)	150 (1.5)	0.36	2175 (1.2)	145 (1.3)	0.75
Atrial fibrillation	24,045 (5.9)	1590 (6.9)	0.01	23,870 (5.9)	1765 (7.3)	<0.001
COPD	57,840 (14.2)	3290 (14.2)	0.99	57,380 (14.1)	3750 (15.4)	0.01
CVA	3995 (1.0)	595 (2.6)	<0.001	3730 (0.9)	860 (3.5)	<0.001
Dementia	6310 (1.5)	205 (0.9)	<0.001	6105 (1.5)	410 (1.7)	0.29
Diabetes	40,910 (10.0)	2210 (9.5)	0.29	40,565 (10.0)	2555 (10.5)	0.25
Heart failure	53,420 (13.1)	3340 (14.4)	0.02	52,655 (13.0)	4105 (16.9)	<0.001
Hypertension	87,930 (21.6)	4690 (20.2)	0.05	86,480 (21.3)	6140 (25.3)	<0.001
Obstructive sleep apnea	26,585 (6.5)	1750 (7.5)	<0.001	26,850 (6.6)	1485 (6.1)	0.19
Mild protein calorie malnutrition	4405 (1.1)	255 (1.1)	0.91	4340 (1.1)	320 (1.3)	0.11
Moderate protein calorie malnutrition	16,370 (4.0)	1200 (5.2)	<0.001	16,080 (3.9)	1490 (6.1)	<0.001
Severe protein calorie malnutrition	24,670 (6.1)	2225 (9.6)	<0.001	24,090 (5.9)	2805 (11.5)	<0.001
Renal disease	48,650 (11.9)	2295 (9.9)	<0.001	47,625 (11.7)	3320 (13.7)	<0.001
Smoking	121,095 (29.7)	5865 (25.7)	<0.001	119,860 (29.5)	7100 (29.2)	0.72
Solid tumor, no metastasis	10,430 (5.9)	550 (5.4)	<0.001	10,215 (5.8)	765 (6.9)	0.03
**BMI Class**			<0.001			0.02
Normal	368,645 (90.4)	20,375 (87.8)		367,005 (90.2)	22,015 (90.6)	
Overweight	1375 (0.3)	50 (0.2)		1350 (0.3)	75 (0.3)	
Class 1 obesity	12,750 (3.1)	875 (3.8)		12,830 (3.1)	795 (3.3)	
Class 2 obesity	9085 (2.2)	610 (2.6)		9315 (2.3)	380 (1.6)	
Class 3 obesity	15,945 (3.9)	1300 (5.6)		16,210 (4.0)	1035 (4.3)	

COPD: chronic obstructive pulmonary disease; CVA: cerebrovascular accident; BMI: body mass index.

**Table 2:** Summary of acute inpatient event rates and secondary outcomes in patients who had venous thromboembolism (VTE) and hemorrhagic events compared to those who did not. Values are presented as absolute counts with percentages in parentheses, representing proportion of encounters within each clinical category with VTE or hemorrhagic events.

Acute Inpatient Events	VTE Absent [407,575 (94.61%)]	VTE Present [23,205 (5.39%)]	*p*-Value	Hemorrhage Absent [406,710 (94.36%)]	Hemorrhage Present [24,300 (5.64%)]	*p*-Value
Acute kidney injury	79,600 (19.5)	6490 (28.0)	<0.001	77,835 (19.1)	8255 (34.0)	<0.001
Acute liver injury	5515 (1.4)	530 (2.3)	<0.001	5260 (1.3)	785 (3.2)	<0.001
Central venous catheter insertion	73,605 (18.1)	7775 (33.4)	<0.001	75,020 (18.5)	6640 (27.3	<0.001
Central venous catheter infection	10,300 (2.5)	1220 (5.3)	<0.001	10,780 (2.7)	740 (3.0)	0.09
Received chemotherapy	96,585 (23.7)	4495 (19.4)	<0.001	99,000 (24.3)	2080 (8.6)	<0.001
Received immunotherapy	2485 (0.6)	105 (0.5)	<0.001	2545 (0.6)	45 (0.2)	<0.001
Invasive mechanical ventilation	12,525 (3.1)	1470 (6.3)	<0.001	10,970 (2.7)	3025 (12.5)	<0.001
Sepsis	73,430 (18.0)	5825 (25.1)	<0.001	72,250 (17.8)	7005 (28.8)	<0.001
Septic shock	21,705 (5.3)	2245 (9.7)	<0.001	2085 (5.1)	3265 (13.4)	<0.001
Thrombocytopenia	95,205 (23.4)	5455 (23.5)	0.81	92,945 (22.9)	7715 (31.8)	<0.001
Thrombocythemia	2410 (0.6)	155 (0.7)	0.51	2410 (0.6)	155 (0.6)	0.69
**Secondary Outcomes**						
Length of stay [Median (interquartile range)], days	5 (3–13)	11.5 (5–27)	<0.001	5 (3–13)	10 (4–26)	<0.001
Cost of care [MD (IQR)], US Dollars	21,363 (9530–57,201)	46,872 (16,473–125,724)	<0.001	21,404 (9541–57,250)	43,663 (15,287–126,279)	<0.001
All-cause inpatient mortality	28,315 (6.9)	2365 (10.2)	<0.001	24,860 (6.1)	5820 (24.0)	<0.001

**Table 3:** Relative risk of venous thromboembolic and hemorrhagic outcomes by malnutrition status (compared to those without protein calorie malnutrition, PCM) using multinomial regression analysis. Base outcome for comparison was Neither outcome (i.e., No VTE and No hemorrhage). KEY: ns = not significant; * *p*-value < 0.05; ** *p*-value < 0.01; *** *p*-value < 0.001.

	Relative Risk Ratio (95% Confidence Intervals)		
Outcome	Mild PCM	Moderate PCM	Severe PCM
VTE Only	1.1 (0.74–1.62) ns	1.27 (1.04–1.54) *	1.28 (1.08–1.51) **
Hemorrhage Only	1.25 (0.85–1.83) ns	1.48 (1.23–1.78) ***	1.73 (1.50–1.99) ***
VTE & Hemorrhage	0.37 (0.05–2.73) ns	2.00 (1.21–3.37) *	2.88 (2.00–4.14) ***

## Data Availability

The raw data supporting the conclusions of this article will be made available by the authors on request.

## Abbreviations

The following abbreviations are used in this manuscript:

MDPI	Multidisciplinary Digital Publishing Institute
DOAJ	Directory of Open Access Journals
HCUP	Healthcare Cost and Utilization Project
TLA	Three-letter acronym
LD	Linear dichroism
DVT	Deep vein thrombosis
VTE	Venous thromboembolism
AHRQ	Agency for Healthcare Research and Quality
NIS	National Inpatient Sample
ICD-10-CM	International Classification of Diseases, Tenth Revision, Clinical Modification
ICD-10-PCS	International Classification of Diseases, Tenth Revision, Procedure Coding System
